# How to draw the line in biomedical research

**DOI:** 10.7554/eLife.00638

**Published:** 2013-03-19

**Authors:** Lisa Huang, Amir Rattner, Han Liu, Jeremy Nathans

**Affiliations:** Department of Molecular Biology and Genetics, Johns Hopkins University School of Medicine, Baltimore, United States and the Department of Biostatistics, Johns Hopkins Bloomberg School of Public Health, Baltimore, United Stateschuang36@jhmi.edu; Department of Molecular Biology and Genetics, Johns Hopkins University School of Medicine, Baltimore, United Statesarattner@jhmi.edu; Department of Biostatistics, Johns Hopkins Bloomberg School of Public Health, Baltimore, United States and the Department of Computer Science, Johns Hopkins University, Baltimore, United States; Current address: Department of Operations Research, Princeton University, Princeton, United States hanliu@princeton.edu; Department of Molecular Biology and Genetics, the Department of Neuroscience, the Department of Ophthalmology and the Howard Hughes Medical Institute, Johns Hopkins University School of Medicine, Baltimore, United Statesjnathans@jhmi.edu

**Keywords:** Tutorial, statistic, publishing

## Abstract

The use of the least squares method to calculate the best-fitting line through a two-dimensional scatter plot typically requires the user to assume that one of the variables depends on the other. However, in many cases the relationship between the two variables is more complex, and it is not valid to say that one variable is independent and the other is dependent. When analysing such data researchers should consider plotting the three regression lines that can be calculated for any two-dimensional scatter plot.

Biomedical research relies on statistical analyses of data sets comprised of multiple variables and, in particular, on analyses of the relationships between pairs of variables within those data sets. In a typical analysis, data representing two variables are displayed in a two-dimensional scatter plot and the method of ordinary least squares is used to fit a regression line to the data. Here we examine an under-appreciated aspect of this approach: the slope of the regression line depends on which of the two variables we select as the independent variable. This means that the method of ordinary least squares can be used to calculate two different regression lines for the same scatter plot. While this issue has long been appreciated in the statistics community, it is not as widely known among biomedical researchers (Cornbleet and Gochman, 1979). The ubiquity of scatter plots and regression lines in biomedical research suggests that a brief discussion of this issue would be useful.

Consider a data set composed of pairs of variables, with individual data points represented by (x_1_, y_1_), (x_2_, y_2_), and so on. In some studies, there may be a symmetric relationship between x_i_ and y_i_: for example, they might represent blood pressure measurements from pairs of siblings in a cohort study. Alternatively, there may be an asymmetry in the relationship between the variables: for example, x_i_ might represent the dose of an antihypertensive drug, and y_i_ might represent the change in blood pressure in a group of subjects treated with various doses of the drug. In this example, drug dose is the independent variable and change in blood pressure is the dependent variable.

Typically, one is interested in determining the most likely value of the dependent variable given the value of the independent one. Thus, in the example described above one might be interested in predicting the change in blood pressure in response to different doses of the drug. However, there are many instances in which it is not clear that one variable depends on the other (independent) variable. For example, individuals with metabolic syndrome have elevated levels of both serum triglyceride and elevated fasting glucose levels: therefore, in a cohort that contains both metabolic syndrome patients and control subjects, one would expect these two variables to be correlated, with a clustering of metabolic syndrome patients at the high end of both distributions (Ford et al., 2002). Although it would be inappropriate to consider one of these variables independent and the other dependent in a mechanistic sense, one might still be interested in calculating the expected level of serum triglycerides given the level of fasting glucose, or the expected level of fasting glucose given the level of serum triglycerides. As described below, estimations in either direction begin with the calculation of a best-fitting regression line.

## The method of least squares

**Figure 1.**
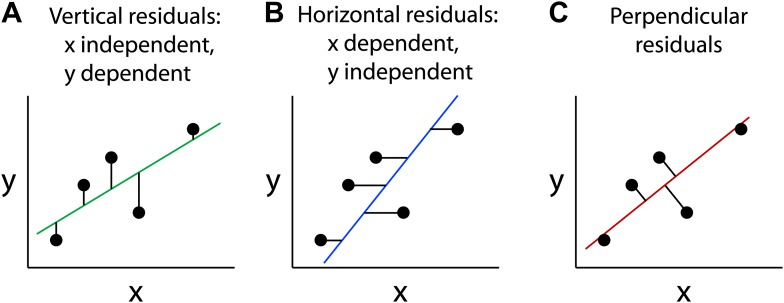
Different types of best-fitting straight lines. These graphs show the best-fitting straight lines through the same five data points as calculated by minimizing the sum of the squares of the vertical residuals, which assumes that x is the independent variable (**A**); horizontal residuals, which assumes that y is the independent variable (**B**); and perpendicular residuals which involves no assumptions about the variables (**C**).

The straight line that constitutes the best fit to a set of data points in the x-y plane is typically calculated by minimizing the sum of the squares of the distances from the points to the line—a method that was introduced by Legendre and Gauss more than two hundred years ago. If one variable, conventionally represented by the y-axis, is known to depend on the other variable, conventionally represented by the x-axis, then one generally calculates a best-fitting line that represents the expected value of the dependent variable (y) as a function of the independent variable (x): this is known as the regression of y on x. In this case, the distance from a data point to the regression line (also known as the residual) is taken as the *vertical* distance from the point to the line (Figure 1A; Bulmer, 1965). This approach, referred to as ordinary least squares regression, is the default mode for line fitting in several commonly used software packages; for example, it is the algorithm represented by the ‘Trend line' and ‘Linest' functions in Microsoft Excel.

If, on the other hand, the y-axis represents the independent variable and the x-axis represents the dependent variable, the best-fitting line can be calculated by taking the residuals as the *horizontal* distances from the points to the line, the regression of x on y (Figure 1B). Except in the limiting case in which all of the data points lie precisely on a straight line, these two best-fitting regression lines will not coincide.

A third type of best-fitting line can be calculated by squaring the *perpendicular* distances from the points to the line (Figure 1C). This method is referred to as an orthogonal or Deming regression. The latter name refers to the statistician W Edward Deming who described the method in the 1940s (Deming, 1943). The Deming regression method is symmetric with respect to the two variables and therefore makes no assumptions regarding dependence and independence (Cornbleet and Gochman, 1979; Linnet, 1993, 1998; Glaister, 2001).

For a scatter plot in which the data points do not fall on a straight line, the best-fitting line calculated with vertical residuals will have a relatively shallow slope (Figure 1A), whereas the best-fitting line calculated with horizontal residuals will have a steeper slope (Figure 1B). The best-fitting line calculated with perpendicular residuals will have an intermediate slope (Figure 1C). The data can also be described by a correlation coefficient (R) that is agnostic with respect to the dependence or independence of the variables (Bulmer, 1965). It is conventional to calculate the square of the correlation coefficient (R^2^), which is equal to the slope of the regression line for y on x (that is, calculated using vertical residuals) divided by the slope of the regression line for x on y (horizontal residuals). Thus, if the data points fall on a straight line (R^2^ = 1), the slopes of these two best-fitting lines will be equal. With increasing scatter in the data (R^2^ < 1), the slopes of the two best-fitting lines will diverge. If the data points are completely uncorrelated (R^2^ = 0), then the best-fitting lines calculated with vertical and horizontal residuals will have slopes of 0 and infinity, respectively.

## Examples with real data: three lines are better than one

**Figure 2.**
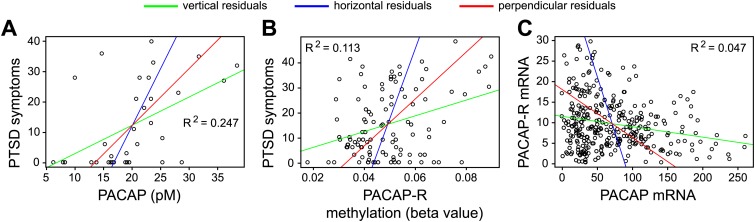
Best-fitting straight lines for three data sets reported by Ressler and co-workers (Ressler et al., 2011). For each of these data sets, best-fitting lines have been calculated by minimizing the sum of the squares of vertical residuals (green), horizontal residuals (blue) or perpendicular residuals (red). The variables in each data set are explained in the text; the data are taken from Figures 1a (**A**), 4a (**B**) and 4c (**C**) in Ressler et al. The agreement between the three lines is relatively poor, as expected from the low values of R^2^, where R is the correlation coefficient. The orthogonal or Deming regression shown by the red lines is not available in Microsoft Excel, but it can be calculated with Excel add-in freeware provided by Jon Peltier (peltiertech.com/WordPress/deming-regression-utility), with the “r” statistics package (www.r-project.org), and with various commercial software packages including Analyse-it (analyse-it.com) and MedCalc (www.medcalc.org/).

These considerations are illustrated by three scatter plots in a paper on post-traumatic stress disorder by Kerry Ressler and co-workers (Ressler et al., 2011). The best-fitting lines for these three plots were calculated with vertical residuals (green lines in Figure 2). In each case, these differ substantially from the best-fitting lines calculated with horizontal residuals (blue lines), as expected from the relatively low values of R^2^ for the three data sets; the best-fitting lines calculated with perpendicular residuals (red lines) occupy intermediate positions.

As noted above, the choice of algorithm to use in calculating the best-fitting line reflects a decision regarding which variable is independent and which is dependent. In Ressler et al. the variables in the three scatter plots are: severity of post-traumatic stress disorder (PTSD) symptoms vs. serum concentration of pituitary adenylate cyclase activating polypeptide (PACAP, also known as ADCYAP1; Figure 2A); severity of PTSD symptoms vs. PACAP receptor gene methylation (also known as ADCYAP1R; Figure 2B); and abundance of PACAP receptor mRNA in the cerebral cortex vs. abundance of PACAP mRNA in the cerebral cortex (Figure 2C). In this example, as in many biological systems, the cause and effect relationships between the variables are likely to be complex. For example, while it is possible that PACAP secretion may alter the severity of PTSD symptoms, it is also possible that stress associated with PTSD may alter PACAP secretion. Furthermore, it is possible that these two variables may have no direct cause-and-effect relationship, and that changes in neural circuitry alter stress level and PACAP secretion by distinct mechanisms. A similar argument can be applied to the other pairs of variables.

Visual inspection of Figure 2 shows that the individual regression lines for y on x (green) or x on y (blue) do not fully capture the trend in the data points within any of the scatter plots. Plotting both regression lines gives a fuller picture of the data, and comparing their slopes provides a simple graphical assessment of the correlation coefficient. Plotting the orthogonal regression line (red) provides additional information because it makes no assumptions about the dependence or independence of the variables; as such, it appears to more accurately describe the trend in the data compared to either of the ordinary least squares regression lines.

## Conclusion

The ordinary least squares method is well suited to the analysis of data sets in which one variable influences or predicts the value of a second variable. In biological systems, where causal relationships between variables are often complex, deciding that one variable depends on the other may be somewhat arbitrary. Moreover, even when a causal chain appears to be well established mechanistically, feedback regulation at the molecular, cellular or organ system level can undermine simple models of dependence and independence. Therefore, we would like to suggest that unless the data analysis calls exclusively for a regression of y on x (or x on y), scatter plots should be presented with three best-fitting lines–calculated with horizontal, vertical and perpendicular residuals–to facilitate a more balanced assessment of trends in the data. Given the ubiquity of the ordinary least squares method in the analysis of two-dimensional scatter plots, this small change in the standard approach to data presentation should prove useful across the full range of biomedical sciences.

## References

[bib1] Bulmer MG (1965). Principles of statistics.

[bib2] Cornbleet PJ, Gochman N (1979). Incorrect least-squares regression coefficients in method-comparison analysis. Clin Chem.

[bib3] Deming WE (1943). Statistical adjustment of data.

[bib4] Ford ES, Giles WH, Dietz WH (2002). Prevalence of metabolic syndrome among US adults: findings from the third National Health and Nutrition Examination Survey. JAMA.

[bib5] Glaister P (2001). Least squares revisited. Mathematical Gazette.

[bib6] Linnet K (1993). Evaluation of regression procedures for methods comparison studies. Clin Chem.

[bib7] Linnet K (1998). Performance of Deming regression analysis in case of misspecified analytical error ratio in method comparison studies. Clin Chem.

[bib8] Ressler KJ, Mercer KB, Bradley B, Jovanovic T, Mahan A, Kerley K (2011). Post-traumatic stress disorder is associated with PACAP and the PAC1 receptor. Nature.

